# Sensitivity and tolerance analysis of single-layer MgF_2_ and SiO_2_ anti-reflection coatings

**DOI:** 10.1038/s41598-025-33607-1

**Published:** 2025-12-22

**Authors:** Erfan Ghapanvari, Mostafa Salahshoor

**Affiliations:** School of Physics, Iran University of Science and Technology, Tehran, 1684613114 Iran

**Keywords:** AR coating, Thin film, Reflectivity, Angle of incidence, Light polarization, Sensitivity analysis, Manufacturing tolerances, Engineering, Materials science, Optics and photonics

## Abstract

Anti-reflection (AR) coatings are integral to enhancing the performance of optical systems. This paper presents a comprehensive simulation-based sensitivity analysis of single-layer magnesium fluoride ($\hbox {MgF}_2$) and silicon dioxide ($\hbox {SiO}_2$) AR coatings on a glass substrate. The investigation focuses on the influence of critical design parameters, namely layer thickness, angle of incidence, and light polarization (s and p), on the spectral reflectance within the visible range. The results demonstrate that the optical performance of these coatings is strongly dependent on the aforementioned parameters. For s-polarized light, reflectance consistently increases with the angle of incidence. In contrast, for p-polarized light, reflectance initially decreases, reaching a minimum near zero at an optimal angle, a phenomenon associated with the Brewster angle. Furthermore, the study reveals that reflectance is highly sensitive to variations in layer thickness, particularly to thickness reduction. Quantitative sensitivity metrics are introduced to evaluate robustness against manufacturing errors. This analysis provides critical insights for the design optimization and the determination of manufacturing tolerances for AR coatings, aimed at maximizing light transmission in practical optical devices.

## Introduction

The control of optical reflection is a fundamental imperative in the design of high-performance optical and photonic systems. Uncontrolled Fresnel reflections at material interfaces induce significant power loss and wavefront distortion, degrading the performance of systems ranging from high-efficiency photovoltaics to diffraction-limited imaging optics and high-power lasers^1,2^. Anti-reflection coatings (ARCs) represent the primary and most effective strategy for mitigating these deleterious effects^3–5^. In photovoltaic applications, ARCs are indispensable for maximizing photon incoupling, thereby enhancing the power conversion efficiency of solar cells^6,7^. For high-energy laser systems, these coatings are critical for minimizing parasitic back-reflections that can induce catastrophic optical damage and limit achievable energy output^8,9^. Similarly, in precision optical instruments, imaging lenses, and displays, ARCs serve to increase transmission, reduce glare, and improve overall image contrast and fidelity^10,11^. The predominant and most versatile approach to realizing AR functionality is through the deposition of meticulously engineered dielectric thin films.

The efficacy of a dielectric ARC is governed by two core principles: the strategic selection of materials with specific refractive indices and the precise control of layer thicknesses to facilitate destructive interference between wavefronts reflected from the film’s top and bottom interfaces^12,13^. Dielectric materials are favored for these applications due to their negligible optical absorption within the operational wavelength band^14^. A conventional multilayer design involves alternating layers of high-refractive-index materials—such as titanium dioxide (TiO$_{\text {2}}$)^4^, hafnium dioxide (HfO$_{\text {2}}$)^15^, or silicon nitride (SiN$_{\text {x}}$)^16^—with low-refractive-index materials. Among the low-index dielectrics, silicon dioxide (SiO$_{\text {2}}$) and magnesium fluoride (MgF$_{\text {2}}$) have long been established as two of the most foundational and widely employed materials in optical thin-film technology^17–20^. While early ARC designs were predicated on single layers optimized for a specific wavelength, contemporary demands for superior performance have driven the evolution toward complex multilayer stacks and nanostructured films engineered to achieve broadband and omnidirectional AR^21–23^. Despite this progression toward greater complexity, the single-layer ARC remains the most fundamental design paradigm and a critical building block for more advanced optical systems.

The single-layer ARC constitutes the simplest and most illustrative implementation of interference-based reflection suppression. Its design is based on the quarter-wavelength optical thickness rule, where a film with an optical thickness (physical thickness multiplied by refractive index) of one-quarter of the target wavelength minimizes reflection at normal incidence. This condition is optimized when the film’s refractive index serves as an optical impedance matching layer between the incident medium and the substrate, ideally satisfying the relation^24,25^

1 $$\begin{aligned} n_{\text {film}} = \sqrt{n_{\text {substrate}} \cdot n_{\text {incident}}} . \end{aligned}$$

Materials such as Magnesium Fluoride (MgF$_{\text {2}}$, $n \approx 1.38$)^26^ and Silicon Dioxide (SiO$_{\text {2}}$, $n \approx 1.45$)^27^ are exceptionally well-suited for this purpose due to their low refractive indices, which effectively bridge the impedance mismatch between air ($n \approx 1$) and common higher-index substrates like glass ($n \approx 1.5$) or silicon^28–30^. Their applications are ubiquitous, ranging from coatings on ophthalmic lenses to performance-enhancing layers on crystalline silicon solar cells^31,32^. However, the performance of single-layer ARCs is intrinsically constrained to a narrow operational bandwidth and exhibits high sensitivity to the angle of incidence^33,34^. It is precisely this high sensitivity to spectral and angular variations that necessitates the detailed, quantitative analysis that forms the basis of the present work^35,36^. However, realizing this theoretical performance is not merely a matter of design; it is fundamentally constrained by the material properties and microstructures imparted by the chosen fabrication methodology.

The deposition technique employed to fabricate an ARC is a critical determinant of its ultimate optical and mechanical characteristics. The choice of fabrication method directly influences key film properties such as density, stoichiometry, microstructure, and surface roughness, which in turn govern the material’s refractive index, mechanical durability, and laser-induced damage threshold^37^. A diverse array of techniques are utilized to deposit MgF$_{\text {2}}$ and SiO$_{\text {2}}$ thin films, categorized broadly as vacuum deposition and solution-based methods. Vacuum deposition techniques, such as electron-beam evaporation^38–40^, physical vapor deposition (PVD)^41^, and sputtering^35^, are mainstays in the production of high-performance optics, offering precise control over film thickness and high purity. In contrast, solution-based/chemical methods provide scalable and often low-cost alternatives. Sol-gel processes are particularly well-suited for large-area applications and allow for the creation of porous films with tunable refractive indices^42,43^. Advanced techniques like plasma-enhanced atomic layer deposition (PEALD)^44^ and laser-induced chemical vapor deposition (LCVD)^27^ enable the growth of highly conformal and uniform layers at low temperatures, making them suitable for sensitive substrates^45,46^. The variability in film properties arising from these distinct fabrication routes underscores the need for a comprehensive understanding of how a coating will perform under real-world operational conditions, particularly concerning its response to external factors such as the angle of incident light.

The dependence of an ARC’s performance on the angle of incidence (AOI) represents a critical limiting factor for numerous applications. A coating optimized for normal incidence ($0^\circ$ AOI) often exhibits significantly degraded performance at oblique angles^10^. This is a pronounced concern for systems such as solar panels, which operate under continuously varying illumination angles, and for optical instruments requiring a wide field of view^6,47^. The performance degradation arises because an increase in AOI lengthens the optical path length within the film. This induces a phase shift that displaces the reflectance minimum from the target wavelength and broadens its spectral profile, particularly for s-polarized light^23^. Consequently, substantial research has been devoted to the development of wide-angle or omnidirectional ARCs^48,49^. Strategies to mitigate angular sensitivity include the design of specific multilayer stacks that maintain low reflectance over a broad angular range^22,50^ and the fabrication of nanostructured surfaces that generate a graded effective refractive index profile^51,52^. A comprehensive performance analysis must, however, extend beyond the angle of incidence to consider other critical parameters, including the polarization state of the incident light.

A complete assessment of ARC performance requires a multi-parameter analysis that encompasses fundamental optical constraints, manufacturing tolerances, and operational viability. For non-normal incidence, the reflectance for s- and p-polarized light diverges, a fundamental optical effect that must be accounted for in polarization-sensitive systems^53,54^. From a manufacturing and process control perspective, the practical efficacy of any design is contingent on fabrication tolerances. Even minor deviations from the optimal quarter-wavelength thickness, often on the scale of nanometers, can significantly impair a coating’s antireflective properties^8,55^. Similarly, residual stress within the film can lead to substrate bowing or delamination, compromising component integrity^44,56^. Finally, operational viability is determined by mechanical and environmental durability. Factors such as abrasion resistance^57,58^ and, for high-power applications, the laser-induced damage threshold (LIDT)^8,59^ are critical determinants of a coating’s real-world utility and lifetime^37,60,61^. The complex interplay among these optical, manufacturing, and durability variables necessitates the use of computational modeling for predictive design, optimization, and sensitivity analysis.

Numerical simulation has become an indispensable tool in the modern design and optimization of optical coatings. Predictive modeling enables researchers to explore vast parameter spaces, evaluate performance trade-offs, and refine designs prior to engaging in costly and time-consuming fabrication^62^. Various computational approaches are employed to analyze ARC performance. Methods such as the finite-difference time-domain (FDTD) are used to solve Maxwell’s equations directly, providing rigorous insights into light interaction with complex nanostructured coatings^21,63^. Ray tracing and matrix-based transfer matrix methods are frequently implemented to simulate the performance of multilayer stacks in solar cells and other optical components^17,64,65^. Furthermore, device-level simulation packages such as PC1D, TCAD, and Silvaco enable detailed analysis of photovoltaic performance, directly linking the optical enhancements afforded by ARCs to improvements in electrical parameters like short-circuit current and power conversion efficiency^66–69^. These simulation studies facilitate the optimization of key parameters such as layer thickness, material composition, and surface geometry to maximize light transmission or absorption^70^. Despite the extensive application of modeling for designing advanced coatings, a focused and systematic sensitivity analysis for the most fundamental single-layer coatings remains a notable gap in the literature.

While extensive research has focused on developing novel multilayer^31,77^, multifunctional^71–73^, and nanostructured^74,75^ ARCs using MgF$_{\text {2}}$ and SiO$_{\text {2}}$, a detailed and systematic sensitivity analysis of the fundamental single-layer coating’s reflectance with respect to its core operational parameters remains foundational yet less explored. The literature is replete with efforts to advance performance by incorporating features such as self-cleaning^76^, mildew resistance^73^, and flexibility^58^, which are often built upon these elementary layers^78,79^. This paper aims to fill this gap by conducting a comprehensive theoretical sensitivity analysis of the reflectance of single-layer MgF$_{\text {2}}$ and SiO$_{\text {2}}$ ARCs. The investigation will systematically quantify the impact of three critical variables: layer thickness, the angle of incidence, and the polarization of incident light. The contribution of this work is to provide a quantitative and fundamental understanding that can guide the design, tolerance specification, and application of these ubiquitous optical coatings in a wide range of practical scenarios, thereby reinforcing the theoretical foundation upon which more complex optical systems are built.

## Theoretical framework and simulation methodology

### Theoretical foundation

The interaction of light with a multilayer thin-film structure is conventionally analyzed using the transfer matrix method^80–82^. In this approach, each layer is represented by a $2 \times 2$ characteristic matrix that connects the tangential components of the electric and magnetic fields at its boundaries. For a single layer situated between a semi-infinite incident medium (air) and a substrate, the total reflectance of the system can be calculated from the concept of optical admittance^80^.

**Fig. 1:**
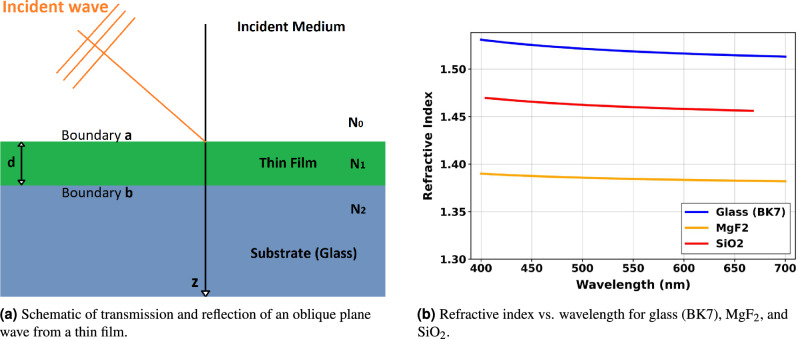
(**a**) Schematic representation of the thin film structure. (**b**) Dispersion of refractive indices for the materials used in this study.

Figure 1aillustrates the reflection and transmission of an obliquely incident plane wave from such a structure. As depicted, the thin film is confined between boundaries a and b. The reflectance (R) of the assembly is given by^80^:

2 $$\begin{aligned} R = \left( \frac{\eta _0 - Y}{\eta _0 + Y}\right) \left( \frac{\eta _0 - Y}{\eta _0 + Y}\right) ^* \end{aligned}$$

where $\eta _0$ represents the tilted optical admittance of the incident medium and *Y* is the effective optical admittance of the structure at the boundary a, which is determined by:

3 $$\begin{aligned} Y = \frac{C}{B} \end{aligned}$$

Here, *B* and *C* are the normalized tangential components of the total electric and magnetic fields at the input interface, respectively. They are calculated using the characteristic matrix of the thin film^80^:

4 $$\begin{aligned} \begin{bmatrix} B \\ C \end{bmatrix} = \begin{bmatrix} \cos \delta _1 & \frac{i\sin \delta _1}{\eta _1} \\ i\eta _1\sin \delta _1 & \cos \delta _1 \end{bmatrix} \begin{bmatrix} 1 \\ \eta _2 \end{bmatrix} \end{aligned}$$

where the parameters are defined as: $E_a, H_a$ is the tangential components of electric and magnetic field amplitudes at boundary a. $E_b, H_b$ is the tangential components of electric and magnetic field amplitudes at boundary b. $\delta _1$ is the phase thickness of the thin film. $\eta _1$ is the tilted optical admittance of the thin film. $\eta _2$ is the tilted optical admittance of the substrate. The optical admittance and phase thickness in Eq. (3) are given by:

5 $$\begin{aligned} \eta _1 = \frac{H_a}{E_a}, \quad \eta _2 = \frac{H_b}{E_b} \end{aligned}$$

6 $$\begin{aligned} \delta _1 = \frac{2\pi N_1 d_1 \cos \vartheta _1}{\lambda } \end{aligned}$$

where $\lambda$ is the free-space wavelength, $N_1$ is the complex refractive index of the thin film, $d_1$ is its physical thickness, and $\vartheta _1$ is the angle of refraction within the film, determined by Snell’s law^80^:

7 $$\begin{aligned} N_0 \sin \vartheta _0 = N_1 \sin \vartheta _1 \end{aligned}$$

Here, $N_0$ is the refractive index of the incident medium (air) and $\vartheta _0$ is the angle of incidence. The tilted optical admittance depends on the polarization of light and is defined for s-polarization (TE) and p-polarization (TM) as follows^80^:

8 $$\begin{aligned} \eta _1&= yN_1\cos \vartheta _1 \quad (\text {s-polarization}) \end{aligned}$$

9 $$\begin{aligned} \eta _2&= yN_2\cos \vartheta _2 \quad (\text {s-polarization}) \end{aligned}$$

10 $$\begin{aligned} \eta _1&= yN_1/\cos \vartheta _1 \quad (\text {p-polarization}) \end{aligned}$$

11 $$\begin{aligned} \eta _2&= yN_2/\cos \vartheta _2 \quad (\text {p-polarization}) \end{aligned}$$

where *y* is the optical admittance of free space ($2.6544 \times 10^{-3}$ Siemens). These relations provide the mathematical foundation for calculating reflectance for arbitrary angles and polarizations.

### Simulation methodology

All optical simulations in this study were performed using the transfer matrix method (TMM) implemented in the Essential Macleod thin-film design software (version 10.2.496)^80^. The model assumes planar, homogeneous, and isotropic layers with perfectly smooth interfaces. The incident medium is air ($N_0 = 1.0$), and the substrate is semi-infinite glass ($N_2 \approx 1.52$). Material dispersion was fully accounted for using the software’s built-in database of optical constants, which provides wavelength-dependent refractive indices for $\hbox {MgF}_2$, $\hbox {SiO}_2$, and glass across the visible spectrum (400–700 nm). These values are consistent with standard references^80–82^. Figure 1bshows the variation of refractive index with wavelength for the three materials used in this study. The reflectance was calculated for both s- and p-polarizations across a range of incidence angles (0$^\circ$ to 75$^\circ$ in 15$^\circ$ increments) and coating thicknesses (varied around the quarter-wave design point). For unpolarized light, the reflectance was taken as the arithmetic average of the s and p-polarized components: $R_{\text {unpol}} = (R_s + R_p)/2$.

### Sensitivity metrics

To quantify the robustness of the AR coatings against manufacturing errors, we introduce two sensitivity metrics. The first is defined as the absolute derivative of reflectance with respect to thickness:

12 $$\begin{aligned} S_d(\lambda ) = \left| \frac{\partial R(\lambda )}{\partial d_1} \right| \end{aligned}$$

where $R(\lambda )$ is the spectral reflectance and $d_1$ is the physical thickness of the coating. This metric provides a direct measure of how sensitive the optical performance is to thickness variations at each wavelength. Similarly, we define a sensitivity metric with respect to the angle of incidence:

13 $$\begin{aligned} S_\theta (\lambda ) = \left| \frac{\partial R(\lambda )}{\partial \theta _0} \right| \end{aligned}$$

where $\theta _0$ is the angle of incidence. This metric quantifies the angular sensitivity of the coating’s performance.

## Results and discussion

### Reflectance of quarter-wave thickness layers

The thickness of an AR coating is a critical parameter that influences not only its optical performance but also its mechanical and chemical stability. For a single-layer AR coating, the condition for minimum reflectance at normal incidence is achieved when the optical thickness of the layer is a quarter of the design wavelength. This condition ensures that the wave reflected from the air-film interface and the wave reflected from the film-substrate interface are out of phase by $\pi$, leading to destructive interference. To achieve zero reflectance, an additional condition on the refractive index, known as impedance matching, must be met: $N_1 = \sqrt{N_0 N_2}$. For an air-glass interface ($N_0 \approx 1, N_2 \approx 1.52$), the ideal refractive index for the coating is $N_1 \approx 1.23$.

14 $$\begin{aligned} N_1 d_1 = \lambda / 4 \end{aligned}$$

**Fig. 2:**
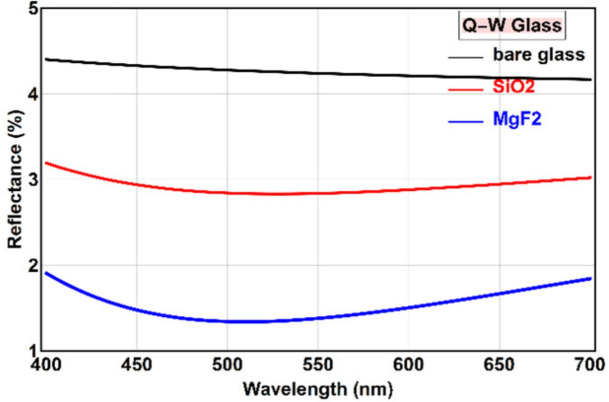
Reflectance versus wavelength for normal incidence on bare glass (black), glass with a quarter-wave $\hbox {MgF}_2$ coating (blue), and glass with a quarter-wave $\hbox {SiO}_2$ coating (red).

Accordingly, the physical quarter-wave thicknesses for $\hbox {MgF}_2$ and $\hbox {SiO}_2$ layers at $\lambda = 510$ nm were calculated to be 92.4 nm and 87.3 nm, respectively. Figure 2 presents the simulated reflectance spectra for these coatings, with the reflectance of uncoated glass provided as a baseline.

The results in Fig. 2 clearly demonstrate the efficacy of the AR coatings. Both materials yield a significant reduction in reflectance across the visible spectrum, with a minimum near the design wavelength of 510 nm. The $\hbox {MgF}_2$ layer reduces the average reflectance by over 50%, while the $\hbox {SiO}_2$ layer achieves a reduction of more than 30%. The superior performance of the $\hbox {MgF}_2$ coating is a direct consequence of its lower refractive index (1.38) being closer to the ideal value of 1.23, thus providing a better impedance match between air and the glass substrate. This leads to a lower minimum reflectance and a broader AR bandwidth.

### Effect of layer thickness variations on reflectance

**Fig. 3:**
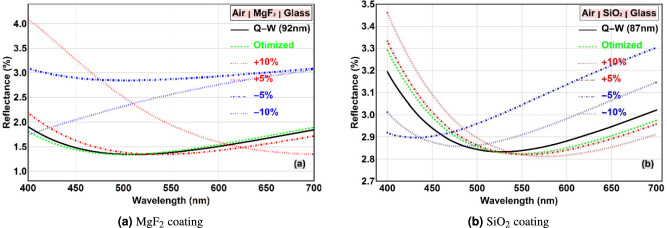
Reflectance versus wavelength for (**a**) $\hbox {MgF}_2$ and (**b**) $\hbox {SiO}_2$ layers with different thicknesses: solid black curve (quarter-wave), red/blue dot-dashed curves ($\pm 5\%$ deviation), red/blue dotted curves ($\pm 10\%$ deviation).

In practical fabrication processes, such as physical vapor deposition, achieving the exact design thickness is challenging, and deviations are inevitable. Therefore, it is crucial to understand the sensitivity of the coating’s performance to such variations. This analysis is essential for establishing acceptable manufacturing tolerances. To this end, we simulated the reflectance for thickness variations of $\pm 5\%$ and $\pm 10\%$ from the ideal quarter-wave thickness. Figure 3 displays these results for both $\hbox {MgF}_2$ and $\hbox {SiO}_2$ layers at normal incidence.

As shown in Fig. 3a, a reduction in the $\hbox {MgF}_2$ layer thickness leads to a significant increase in reflectance, nearly doubling it at some wavelengths for a $-10\%$ deviation. Conversely, a $+5\%$ increase in thickness has a minimal effect, while a $+10\%$ increase raises the reflectance for $\lambda < 600$ nm but slightly decreases it for longer wavelengths. This asymmetry indicates that the coating’s performance is more sensitive to a decrease in thickness than an increase. Physically, a thinner layer fails to provide the necessary $\pi$ phase shift for optimal destructive interference at the design wavelength, causing the reflectance minimum to shift to shorter wavelengths and the overall reflectance to rise. The interference condition is more forgiving to excess material than to insufficient material.

A similar trend is observed for the $\hbox {SiO}_2$ layer in Fig. 3b. An increase in thickness shifts the reflectance minimum to longer wavelengths, while a decrease shifts it to shorter wavelengths. A $10\%$ thickness decrease results in a more pronounced performance degradation across the central visible region compared to a $10\%$ increase. These findings underscore the importance of precise thickness control during deposition, with a particular emphasis on avoiding under-deposition to maintain performance.

### Effect of angle of incidence and polarization on reflectance

**Fig. 4:**
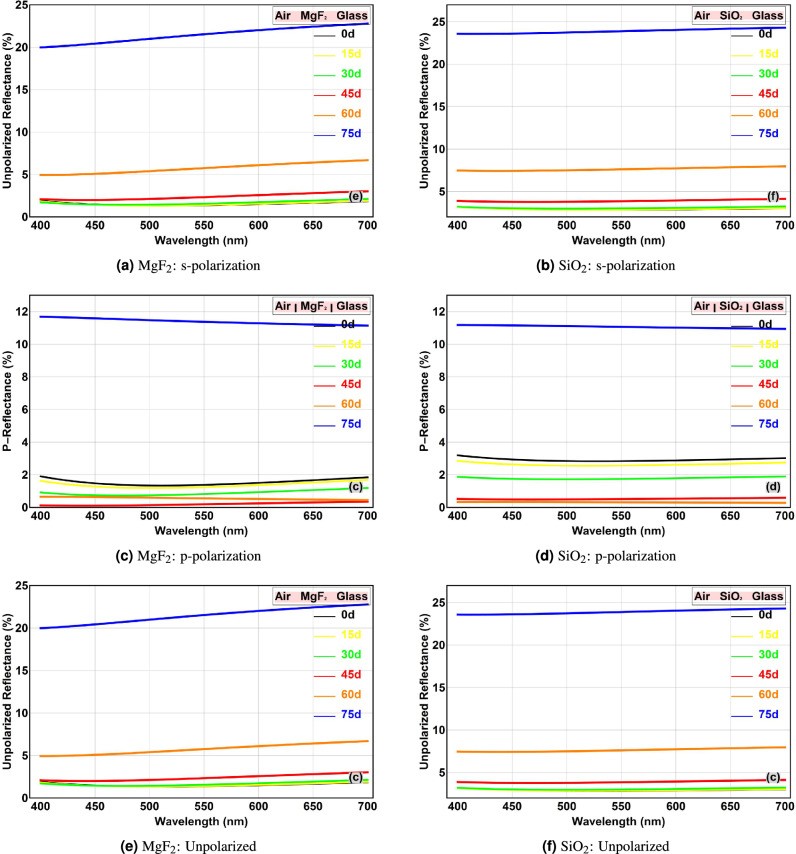
Reflectance versus wavelength at different angles of incidence (0$^\circ$, 15$^\circ$, 30$^\circ$, 45$^\circ$, 60$^\circ$, 75$^\circ$) for: (**a,b**) s-polarization, (**c,d**) p-polarization, and (**e,f**) unpolarized light. Left column: $\hbox {MgF}_2$ coating. Right column: $\hbox {SiO}_2$ coating.

The performance of AR coatings is inherently dependent on the angle of incidence, as the optical path length within the film and the effective admittance at the interfaces change with angle. Figure 4 presents a comprehensive view of the angular and polarization dependence for both $\hbox {MgF}_2$ and $\hbox {SiO}_2$ coatings.

For s-polarization (Fig. 4aand 4b), the reflectance for both coatings systematically increases with the angle of incidence. This is a characteristic behavior for TE waves at a dielectric interface, as the Fresnel reflection coefficient for s-polarization increases monotonically with angle due to the increasing impedance mismatch. The increase is moderate for angles up to 45 degrees but becomes substantial at larger angles, posing a limitation for wide-angle applications where unpolarized or s-polarized light is present.

In stark contrast, p-polarization exhibits a markedly different behavior, as seen in Fig. 4cand 4d. For both coatings, as the angle of incidence increases, the reflectance initially decreases, reaching a value close to zero at a specific optimal angle, before increasing again at even larger angles. This phenomenon is a manifestation of the Brewster’s angle effect, at which p-polarized light experiences perfect transmission (zero reflection) when the reflected and refracted rays are perpendicular. For the $\hbox {MgF}_2$ and $\hbox {SiO}_2$ coatings on glass, the presence of the thin film modifies this condition, resulting in a ”pseudo-Brewster angle” where reflectance is minimized. For the $\hbox {MgF}_2$ and $\hbox {SiO}_2$ systems, this optimal angle is approximately 45 and 60 degrees, respectively. This unique property can be exploited in applications requiring high transmission of p-polarized light, such as in polarizers or specific laser systems.

The averaged spectra for unpolarized light (Fig. 4eand 4f) demonstrate intermediate behavior between the two polarization states. At small angles ($<30^\circ$), the reflectance remains low across the visible spectrum. At larger angles, the rapid rise in $R_s$ dominates the average, leading to increased reflectance. This highlights the challenge of designing wide-angle AR coatings for applications with unpolarized illumination.

### Contour analysis of reflectance versus thickness and angle

**Fig. 5:**
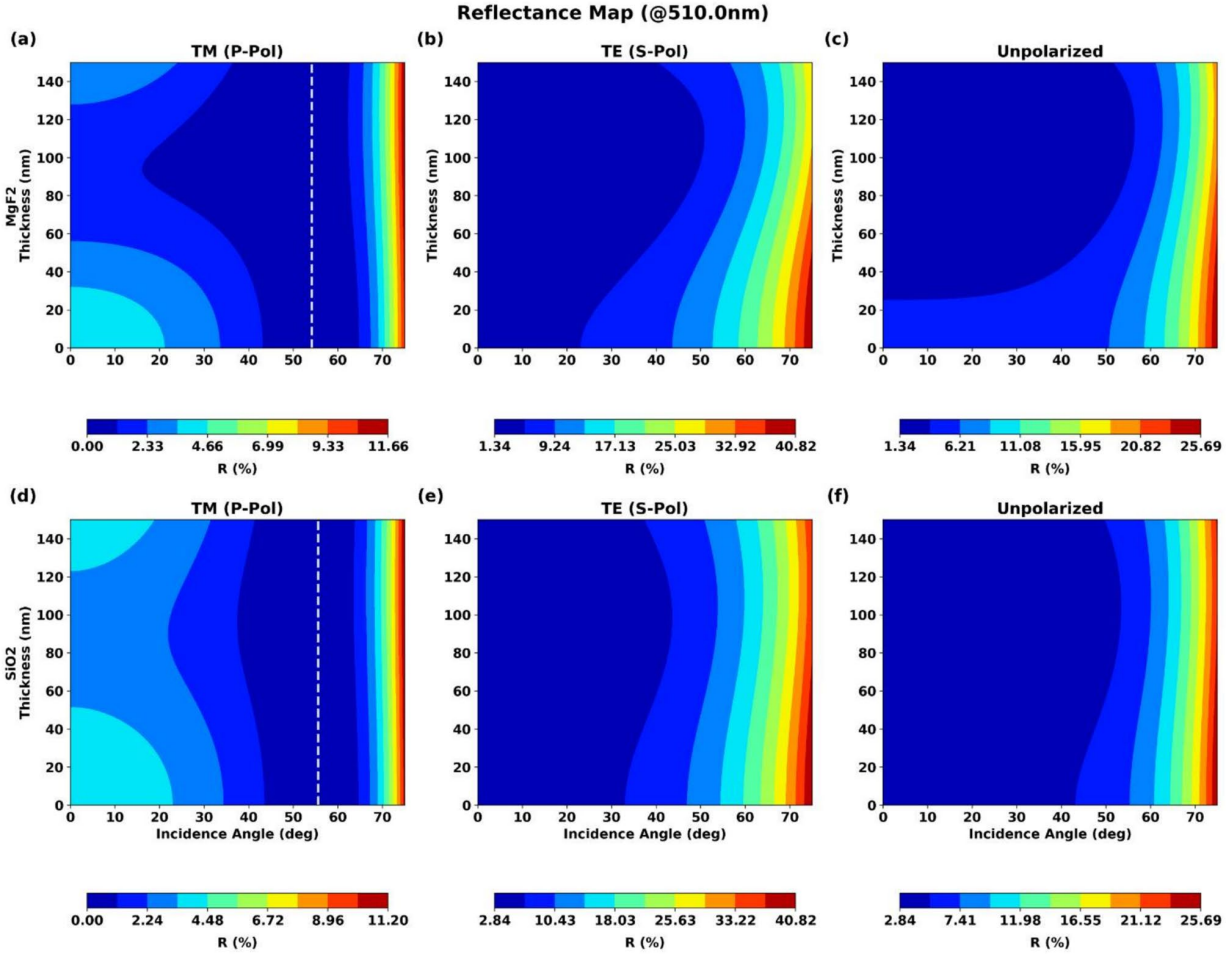
2D contour plots of reflectance versus coating thickness and angle of incidence for $\hbox {MgF}_2$ and $\hbox {SiO}_2$ coatings for (**a**) s-polarization, (**b**) p-polarization, and (**c**) unpolarized light.

To gain a more holistic understanding of the design space, we analyzed the combined influence of thickness and angle of incidence on reflectance at the central wavelength (510 nm). The two-dimensional contour plots in Fig. 5 provide comprehensive performance maps for both materials and all three polarization states.

The plots in Fig. 5 reveal distinct behaviors for different polarization states. For s-polarization, the region of lowest reflectance (dark blue) is confined to small angles of incidence (typically < 30 degrees) regardless of the layer thickness. The contours are nearly vertical, indicating that reflectance is more strongly dependent on the angle of incidence than on minor thickness variations in this regime. The reflectance deteriorates rapidly as the angle increases, reinforcing the challenge of designing wide-angle AR coatings for s-polarized light.

The design space for p-polarization is significantly different and offers more flexibility. There exists a distinct ”valley” of near-zero reflectance (dark blue) corresponding to the Brewster angle condition. For the $\hbox {MgF}_2$ coating, a broad optimal region exists for thicknesses around 90 nm and angles between 20 and 60 degrees. For the $\hbox {SiO}_2$ coating, the optimal region is centered around a thickness of 100 nm and angles between 40 and 60 degrees. This indicates that for p-polarized applications, a more robust design with greater tolerance to angular and thickness variations can be achieved by targeting this operational sweet spot.

For unpolarized light, the contour plots show intermediate behavior, with the lowest reflectance regions lying between those of s- and p-polarizations. This provides a practical guide for applications with natural illumination where light is typically unpolarized.

### Brewster angle analysis

**Fig. 6:**
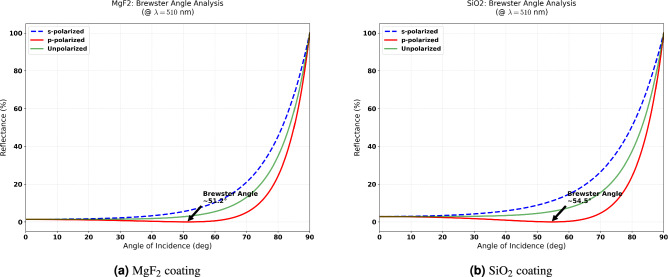
Brewster angle analysis at $\lambda = 510$ nm for (**a**) $\hbox {MgF}_2$ and (**b**) $\hbox {SiO}_2$ coatings. The pseudo-Brewster angles where p-polarized reflectance is minimized are approximately 51.2$^\circ$ and 54.5$^\circ$, respectively.

To further investigate the polarization-dependent behavior, we analyzed the reflectance at the fixed design wavelength (510 nm) as a function of incidence angle for both polarizations. Figure 6 shows the detailed Brewster angle analysis.

The bare glass-air interface has a Brewster angle of $\theta _B = \arctan (1.52) \approx 56.7^\circ$. The presence of the thin film shifts this condition, resulting in pseudo-Brewster angles of approximately 51.2$^\circ$ for $\hbox {MgF}_2$ and 54.5$^\circ$ for $\hbox {SiO}_2$. At these angles, p-polarized reflectance approaches zero, while s-polarized reflectance continues to increase. The unpolarized reflectance shows an intermediate minimum, but remains significantly higher than the p-polarized minimum.

### Sensitivity contour analysis

**Fig. 7:**
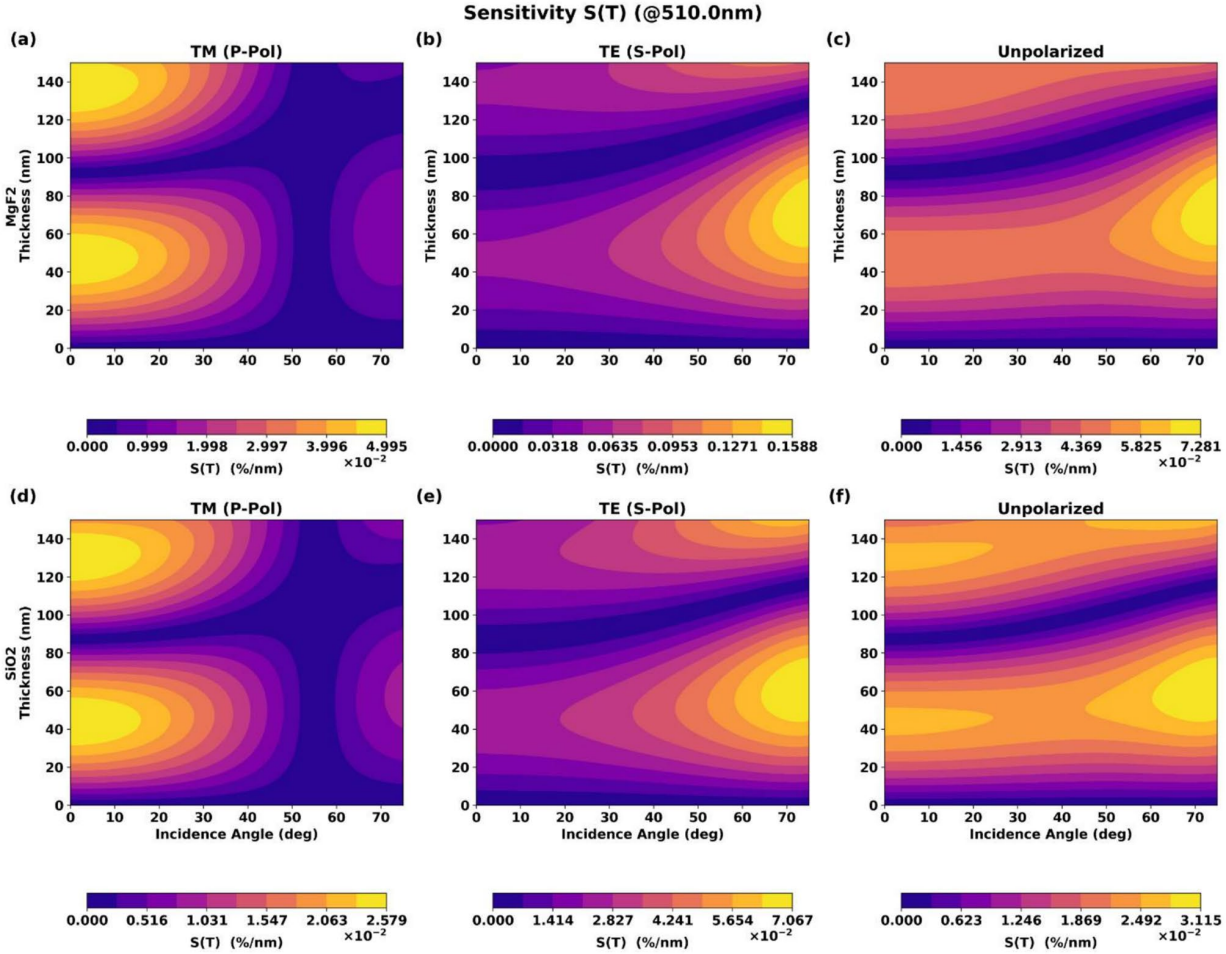
2D contour plots of thickness sensitivity $S_d(\lambda )$ versus coating thickness and angle of incidence for $\hbox {MgF}_2$ and $\hbox {SiO}_2$ coatings for (**a**) s-polarization, (**b**) p-polarization, and (**c**) unpolarized light.

To quantitatively assess the robustness of the coatings against manufacturing and operational errors, we calculated the sensitivity metrics $S_d(\lambda )$ and $S_\theta (\lambda )$ defined in Eqs. (11) and (12). Figure 7 shows the contour plots of thickness sensitivity for both materials and all three polarization states.

The results demonstrate that sensitivity to thickness variations is minimized at the design wavelength (510 nm) and near the optimal operating conditions. For both materials, sensitivity increases at shorter and longer wavelengths, indicating that broadband performance is more susceptible to thickness variations. $\hbox {MgF}_2$ exhibits slightly lower sensitivity than $\hbox {SiO}_2$ near the design wavelength, consistent with its better impedance matching.

**Fig. 8:**
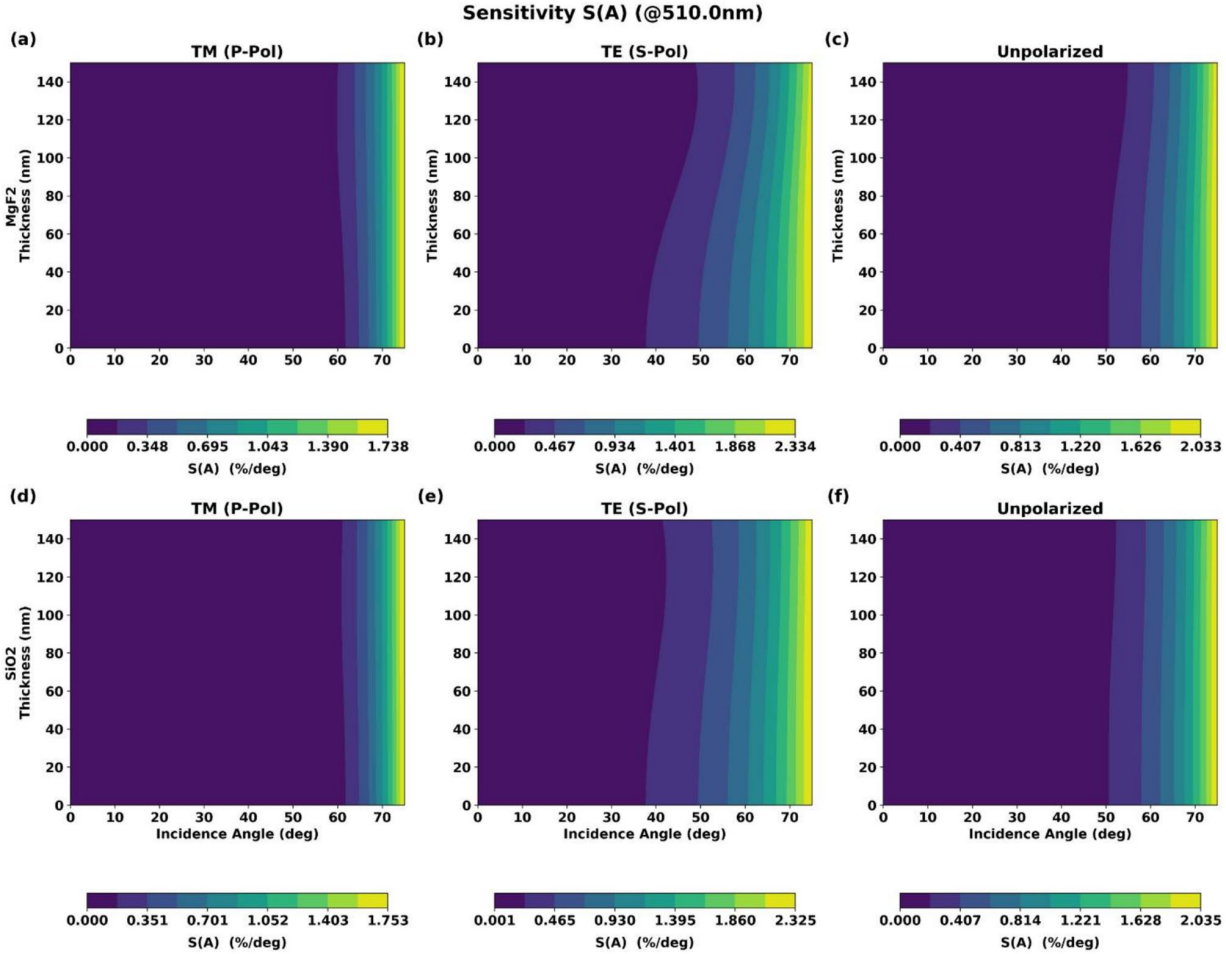
2D contour plots of angular sensitivity $S_\theta (\lambda )$ versus coating thickness and angle of incidence for $\hbox {MgF}_2$ and $\hbox {SiO}_2$ coatings for (**a**) s-polarization, (**b**) p-polarization, and (**c**) unpolarized light.

Figure 8 shows the corresponding contour plots for angular sensitivity $S_\theta (\lambda )$.

The angular sensitivity analysis reveals that for s-polarization, sensitivity increases monotonically with angle, consistent with the observed reflectance behavior. For p-polarization, sensitivity is minimized near the pseudo-Brewster angle, where reflectance changes slowly with angle. This provides valuable insight for designing robust AR coatings for specific angular applications.

### Fabrication considerations and material selection

Single-layer $\hbox {MgF}_2$ and $\hbox {SiO}_2$ coatings are typically deposited using physical vapor deposition (PVD) techniques such as electron-beam evaporation or sputtering. These methods allow precise thickness control at the nanometer scale, which is critical given the sensitivity to thickness variations demonstrated in this study. In-situ optical monitoring during deposition is often employed to achieve the required precision. The choice between $\hbox {MgF}_2$ and $\hbox {SiO}_2$ involves trade-offs between optical performance and practical considerations. MgF$_2$ offers superior optical performance due to its lower refractive index (closer to the ideal $\sqrt{1.52} \approx 1.23$), resulting in lower minimum reflectance and broader bandwidth. However, $\hbox {MgF}_2$ films are relatively brittle and can be more susceptible to mechanical stress and environmental degradation (e.g., humidity-induced microcracking). $\hbox {SiO}_2$ has slightly higher refractive index, leading to somewhat higher residual reflectance. However, $\hbox {SiO}_2$ offers excellent chemical stability, mechanical robustness, and resistance to humidity and temperature cycling. It is often preferred for applications requiring long-term durability in harsh environments. For applications where maximum transmission efficiency is paramount (e.g., high-performance optics, laser systems), $\hbox {MgF}_2$ is the preferred choice. For applications requiring durability, environmental stability, and cost-effectiveness (e.g., consumer optics, outdoor solar cells), $\hbox {SiO}_2$ may be more appropriate despite its slightly lower optical performance.

## Limitations and future work

This study was conducted under idealized conditions to isolate the fundamental sensitivity of AR coatings to key design parameters. Several practical factors were not included. Surface roughness and scattering losses were neglected. Real coatings exhibit some degree of roughness, which can increase diffuse reflection and affect polarization-dependent behavior. Furthermore, temperature-dependent effects such as refractive index drift and thermal expansion were not considered. These factors can be important in applications exposed to significant temperature variations. Additionally, manufacturing defects including thickness non-uniformity, pinholes, and contamination were not modeled. Such defects can degrade performance beyond the idealized sensitivity predictions. Moreover, the analysis was limited to single-layer coatings on glass substrates. Multilayer designs and other substrate materials would require separate investigation.

Future work could extend this analysis to include these practical factors, experimentally validate the sensitivity predictions, and investigate multilayer designs. The methodology developed here–combining transfer matrix simulations with quantitative sensitivity metrics–provides a foundation for such investigations.

## Conclusion

This study presented a detailed sensitivity analysis of single-layer $\hbox {MgF}_2$ and $\hbox {SiO}_2$ AR coatings, investigating the impact of layer thickness, angle of incidence, and light polarization on their reflectance. The simulation results quantitatively demonstrate that the performance of these fundamental optical components is highly sensitive to their design and operating parameters.

The key findings are threefold. First, deviations from the optimal quarter-wave thickness can significantly degrade anti-reflective performance, with the system showing greater sensitivity to a reduction in thickness. Quantitative sensitivity metrics confirmed that the quarter-wave design minimizes sensitivity at the design wavelength, providing maximum robustness against manufacturing errors. Second, the angular response of the coatings is strongly polarization-dependent. While reflectance for s-polarization monotonically increases with the angle of incidence, p-polarization exhibits a reflectance minimum close to zero at the pseudo-Brewster angle. This phenomenon offers a valuable opportunity for designing highly efficient optical components for p-polarized light. Third, the combined analysis of thickness and angle reveals that for p-polarization, a broad design window exists where low reflectance can be maintained, allowing for more robust and fault-tolerant designs.

The practical implications of this analysis are significant. For manufacturers, the sensitivity results provide clear guidance on acceptable thickness tolerances. For designers, the contour maps identify optimal operating regions for specific applications. The comparison between $\hbox {MgF}_2$ and $\hbox {SiO}_2$ helps in material selection based on whether optical performance or environmental durability is prioritized.

In summary, the effective design of an AR coating requires careful consideration of the specific application, including the expected range of incidence angles and the polarization state of the light. The results of this work provide a practical and quantitative guide for optical engineers and manufacturers to optimize the performance of AR coatings, thereby enhancing the efficiency of light transmission in a wide array of optical systems.

## Data Availability

All data generated or analysed during this study are included in this published article.
